# Impact of digital inclusive finance on agricultural total factor productivity in Zhejiang Province from the perspective of integrated development of rural industries

**DOI:** 10.1371/journal.pone.0298034

**Published:** 2024-04-05

**Authors:** Shasha Jin, Zhangqi Zhong

**Affiliations:** 1 School of Finance and Accounting, Zhejiang Institute of Economics and Trade, Hangzhou, Zhejiang, China; 2 School of Economics and Trade, Guangdong University of Foreign Studies, Guangzhou, Guangdong, China; Guangzhou Institute of Geography, Guangdong Academy of Sciences, CHINA

## Abstract

Improving agricultural total factor productivity is crucial for comprehensive rural revitalization and building a strong agricultural nation. Digital inclusive finance amalgamates the benefits of digital technology and inclusive finance, mitigating financial exclusion in agricultural production. It fosters rural revitalization and the modernization of agriculture by bolstering farmers’ innovation, entrepreneurship, and agricultural technology advancements. Consequently, it significantly enhances overall agricultural total factor productivity. This study uses panel data from 2011 to 2020 to empirically investigate the impact and mechanism of digital inclusive finance on agricultural total factor productivity in Zhejiang Province, China. The research results show that digital inclusive finance significantly enhances agricultural total factor productivity in Zhejiang Province, which holds true even after a series of robustness tests. Analysis of the mechanism reveals that the integrated development of rural industries plays a crucial mediating role in empowering agricultural total factor productivity through digital inclusive finance. Furthermore, heterogeneity analysis indicates that the positive effect of digital inclusive finance on agricultural total factor productivity is more pronounced in the northeastern region of Zhejiang Province and in areas ranked in the second tier of agricultural development. Therefore, we recommend comprehensively enhancing the development of digital inclusive finance in rural areas, fostering a financial ecosystem that integrates rural industries, promoting the coordinated development of digital inclusive finance in different regions, and comprehensively improving agricultural total factor productivity.

## Introduction

China is a major agricultural nation, as articulated in the report of the 20th National Congress of the Communist Party of China. This report emphasizes the comprehensive advancement of rural revitalization and the accelerated development of an agricultural powerhouse. The concept of an agricultural powerhouse is the foundation of a socialist modernization drive. The 2023 Central Document No. 1 of China defines an agricultural powerhouse in terms of robust supply assurance, advanced technological equipment, strong operational systems, resilient industries, and competitive capabilities. A pivotal indicator is the agricultural total factor productivity (TFP), which is a comprehensive measure of the overall efficiency of the agricultural production system. It reflects the technological advancement in agricultural economics and assesses the level of agricultural development. This metric effectively encapsulates the core features of an agricultural powerhouse. Therefore, developing an agricultural powerhouse requires a holistic improvement in agricultural TFP.

Finance is the lifeblood of the real economy and constitutes a pivotal pillar for rural revitalization and the development of an agricultural powerhouse. Historically, financing for resolving China’s “three rural issues” (agriculture, rural areas, and farmers) has been characterized by difficulties in access, high costs, and delays, which hinder the sustained growth of agricultural TFP. Technological empowerment has become a significant driver in expanding the coverage and accessibility of rural financial services. Digital inclusive finance is a new financial format driven by technology; it was proposed in 2016 at the G20 Hangzhou Summit and since has noticeably promoted innovation within financial institutions, substantially increased agricultural products and services, and significantly alleviated problems related to low accessibility, high costs, and elevated risks in rural finance.

As one of China’s most economically developed provinces, Zhejiang has long been at the forefront of agriculture. Zhejiang’s rural initiatives are perceived nationwide to have distinct policy advantages, given four unique designations: Zhejiang is the “Demonstration Zone for High-Quality Development and Common Prosperity Construction,” the “Provincial-Level Collaborative Province for Rural Revitalization,” the “Digital Rural Pacesetter,” and the “Pioneer Zone for the Promotion of Small-Scale Agricultural Machinery in Hilly and Mountainous Areas.” Fruitful achievements in rural development have placed Zhejiang at the forefront of the effort to establish China as an agricultural powerhouse. As a technologically advanced province, Zhejiang has rapidly expanded the scope of digital inclusive finance through technological means. According to data from the Ant Group’s online bank, digital financial services reached all 26 counties in Zhejiang’s mountainous regions as of October 2021. By the end of the same year, over two million county-level micro-entrepreneurs and farmers in Zhejiang had accessed digital loans, placing the province at the forefront of county-level digital lending across China. Given Zhejiang’s leading position in both digital inclusive finance and agriculture, the question arises of whether a clear synergy exists between the two, which is a topic that merits in-depth exploration. The mechanisms through which Zhejiang’s digital inclusive finance system influences agricultural development warrant further investigation. Whether from a theoretical or empirical perspective, a clear understanding of the impact and mechanisms by which digital inclusive finance, as evaluated by agricultural TFP, affects agricultural development is vital. Such knowledge can help government agencies and financial institutions improve rural financial services and formulate supportive agricultural financial policies. This research is vital for promoting areas and countries hoping to revitalize their rural zones and strengthen agricultural construction.

## Literature review

In the existing research, the impact of digital inclusive finance on the agriculture-related field is viewed from three viewpoints: farmers, rural areas, and agriculture. It significantly impacts farmers by enhancing their living standards [1, 2], improving access to credit [3], reducing the vulnerability to poverty [4], fostering entrepreneurship among rural residents [5], and consequently, narrowing the income gap between urban and rural areas [6]. In rural areas, digital inclusive finance has extended its reach to vast rural and remote regions by providing expedited financial services through mobile payments and settlement methods. This expansion has significantly enhanced the financial system’s capacity to serve rural markets [7]. Empowering rural finance through digital technology not only optimizes the efficiency of financial resource allocation and stimulates endogenous motivation for the supply side but also fosters the sustainable and balanced development of inclusive finance in rural areas [8]. Furthermore, by fostering technological innovation and increasing the penetration of technology in industries, digital inclusive finance has provided a new impetus for the integrated development of rural industries [9]. Digital inclusive finance enhances agricultural mechanization by increasing farmer income and promoting fixed-asset investments [10]. It also promotes agricultural modernization through local economic development, financial access, business innovation, and financial support for agriculture [11]. Adegbite and Machethe(2020) [12] indicate that digital financial institutions and gender responsive agricultural finance innovations play an important role in enhancing the role of small-scale agriculture in sustainable development in Nigeria.

The growth of agricultural TFP is a foundation for agricultural economic growth and the high-quality development of agriculture. The academic community has intensely studied how to measure agricultural TFP and its influencing factors. Existing research has primarily focused on methods to calculate agricultural TFP. Lin (1992) [13] used the Cobb–Douglas production function method, Fan (1997) [14] used growth accounting, Yu et al. (2011) [15] applied stochastic frontier analysis, and Hou et al. (2012) [16] used the data envelopment analysis–based (DEA-based) Malmquist index method. Gao (2015) [17] argued that TFP measurement methods offer no “absolute best” tool and that the DEA-Malmquist index method, which does not require pre-specification of a production frontier, possesses a “comparative advantage” over other methods, leading to a more comprehensive understanding of the growth patterns of Chinese agriculture. Studies have indicated that various factors, such as the position of agriculture in the overall economy, the degree of agricultural marketization, the level of economic development, farmer income levels, financial support for agriculture, agricultural structure, and resource utilization [18, 19] affect the growth of China’s agricultural TFP in different ways.

Recently, scholars have focused on how models and mechanisms of digital inclusive finance development affect agricultural TFP. Zheng and Li(2022) [20] analyzed Chinese county-level panel data from 2014 to 2018 by using an unconditional quantile fixed-effects model. The results indicate that the development of digital inclusive finance significantly promotes the growth of agricultural TFP at the county level, with the magnitude of the impact depending on the relative productivity of the counties. Moreover, Ren and Lei (2022) [21] used data from 30 Chinese provinces from 2011 to 2020 to empirically demonstrate through a mediation-effects model that digital inclusive finance directly affects agricultural TFP. Additionally, increasing agricultural capital acts as an intermediary between digital inclusive finance and agricultural TFP, primarily realized through technological progress rather than through efficient agricultural technology. This effect is more pronounced in areas with large-scale cultivation and in the central region. Some studies analyzed provincial panel data in China and confirmed that digital inclusive finance drives the growth of green agricultural TFP. They reported that digital inclusive finance enhances agricultural green TFP by optimizing the structure of the agricultural industry [22], stimulating agricultural technological innovation [23, 24], and promoting the marketization of rural land transfer [25].

To summarize, existing research has obtained significant results regarding the theoretical mechanisms and empirical analyses of how digital inclusive finance influences agricultural development. These findings are valuable and serve as a foundation for this study. However, most studies on agricultural TFP have focused on nationwide provincial-level data, which limits their ability to accurately address issues related to imbalanced development between prefecture-level cities within provinces [26]. Given this situation, this paper leverages the advantage of digital inclusive finance index data at various administrative levels to expand the research on the relationship between digital inclusive finance and agricultural productivity to the level of prefecture cities, which has significant practical relevance. Furthermore, digital inclusive finance development, the integrated development of rural industries, and agricultural development vary significantly between different regions in China, with distinct foundations, emphases, and developmental patterns. Therefore, researching on a national scale would face challenges related to variable selection, data acquisition, and the interpretability of the research results [27]. Zhejiang is a digital innovation hub and a province known for its agriculture; it is a benchmark and exemplar in both digital inclusive finance and agricultural development nationwide. This paper thus uses Zhejiang as a case study to investigate how digital inclusive finance affects agricultural TFP at the level of prefecture cities. It offers valuable insights for understanding the disparities in development among cities within provinces in other regions.

Given these considerations, this paper first analyzes in detail data from prefecture-level cities in Zhejiang to investigate how digital inclusive finance affects agricultural TFP and its mechanisms and heterogeneity. This research enriches the empirical evidence at the level of prefecture cities in leading provinces. Second, rural industry integration is a crucial driving factor for agricultural development. This paper constructs a comprehensive assessment index that reflects the degree of rural industry integration and uses this index as an intermediary mechanismto examine how the degree of rural industry integration affects the impact of digital inclusive finance on agricultural TFP. These results provide new theoretical and practical foundations for evaluating the mechanisms through which digital inclusive finance contributes to developing an agricultural powerhouse. Third, based on the empirical results from Zhejiang’s prefecture-level cities, this paper proposes strategies and recommendations for how digital inclusive finance can better serve the goals of comprehensive rural revitalization and the development of an agricultural powerhouse.

## Theoretical mechanisms and research hypotheses

### Direct effects of digital inclusive finance on agricultural total factor productivity

Advancements in digital technology, such as the internet and big data, have empowered inclusive finance to transcend temporal and spatial constraints. This development has enabled low-cost, high-efficiency fund matching to some extent, addressing the shortcomings of traditional financial services. From the supply-side perspective, it has enhanced the accessibility of financial services in agricultural sectors [28], providing ample capital elements for agricultural development and rural revitalization. Digital inclusive finance may directly affect agricultural development through its inherent characteristics and advantages.

First, digital inclusive finance supports rural entrepreneurship and innovation, promoting agricultural development. It facilitates reaching rural underserved micro-entrepreneurs and small-scale farmers, catering more effectively to their decentralized and small-scale financial needs. It allows for rapid risk assessment of rural financial subjects and the design of suitable credit products, making available low-cost financial support for rural entrepreneurs and thereby boosting entrepreneurial activity in rural areas [29, 30]. Rural entrepreneurship not only contributes to local development but also fosters unique, high-quality local brands. By developing businesses, improving the value chain, driving rural employment, and helping farmers increase production and income, digital inclusive finance addresses the challenges of agricultural economic development. Additionally, it uses idle arable land for crop production, attracting various investments in agriculture and enhancing agricultural quality.

Second, digital inclusive finance supports agricultural technological innovation, thereby modernizing agriculture. It increases the agricultural funds and fixed-asset investments in agriculture, particularly for acquiring agricultural machinery. Consequently, this raises the level of agricultural mechanization and ultimately modernizes agricultural production [10]. Digital inclusive finance promotes innovation in agricultural technology by increasing investment in research and development of agricultural technology. In turn, the widespread application of digital smart devices and technologies is promoted in agriculture, enabling real-time monitoring and precise control of agricultural production. This process enhances the level of agricultural modernization [31], which increases the agricultural TFP. Based on these premises, we propose the following research hypothesis:

H1: Digital inclusive finance significantly promotes the enhancement of agricultural TFP.

### Transmission to digital inclusive finance for enhancing agricultural total factor productivity

Rural industry integration refers to a recent agricultural organizational model formed by integrating and cross-border restructuring of agriculture with secondary and tertiary industries, innovating agricultural business models, and modernizing agriculture [32]. Digital inclusive finance promotes rural infrastructure development, providing various digital financial services such as mobile payments, online lending, and insurance to rural households and offering full-service “e-commerce plus finance” solutions for agricultural producers [10]. This approach supports the growth and expansion of new agricultural business entities [33]. Additionally, it leverages e-commerce platforms to improve the standardization of agricultural products and expand sales channels, thereby enhancing the level of rural industry integration. Digital inclusive finance mitigates information asymmetry, breaking traditional credit constraints and financial exclusion. It broadens the boundaries of financial services, enhances the accessibility of financial resources, diversifies financial services, and answers the multilevel financing needs of rural industry integration [34, 35]. It also optimizes agricultural technological equipment and improves the efficiency of financial services, gradually becoming a vital support for the development of rural industry integration.

Rural industry integration further enhances agricultural TFP by strengthening innovation in agricultural technology, agricultural business systems, and agricultural industry resilience, contributing to the construction of an agricultural powerhouse. First, rural industry integration helps overcome the dilemma of single-industry development, proposes new concepts and ideas for cross-industry development, and catalyzes new technological developments, all of which leads to innovation and progress in agricultural technology [36, 37]. In addition, the integration of rural industry promotes the rapid development of new rural business models, which helps to construct a new agricultural business system. Using system dynamics to simulate different support policies, Cao et al. (2022) [38] found that the significant growth of new rural industries such as leisure agriculture, rural tourism, and rural e-commerce promotes rural revitalization, optimizes agricultural business systems, and advances agricultural development. Furthermore, rural industry integration aims to achieve the cross-border integration of various elements such as resources, factors, technology, and market demand in rural areas, becoming a new driving force for ensuring food security, increasing farmer income, technological innovation, and environmental protection. It optimizes the spatial layout of rural industries, guides the aggregation of rural resources, stimulates entrepreneurial and innovative vitality in rural areas, develops industries, improves the resilience of agricultural industries, and lays the foundation for sustainable agriculture development [39]. Therefore, rural industry integration is vital for enhancing agricultural TFP and a key factor for constructing an agricultural powerhouse. Based on these considerations, we propose the following research hypothesis:

H2: Digital inclusive finance enhances agricultural TFP by promoting rural industry integration.

**Fig 1:**
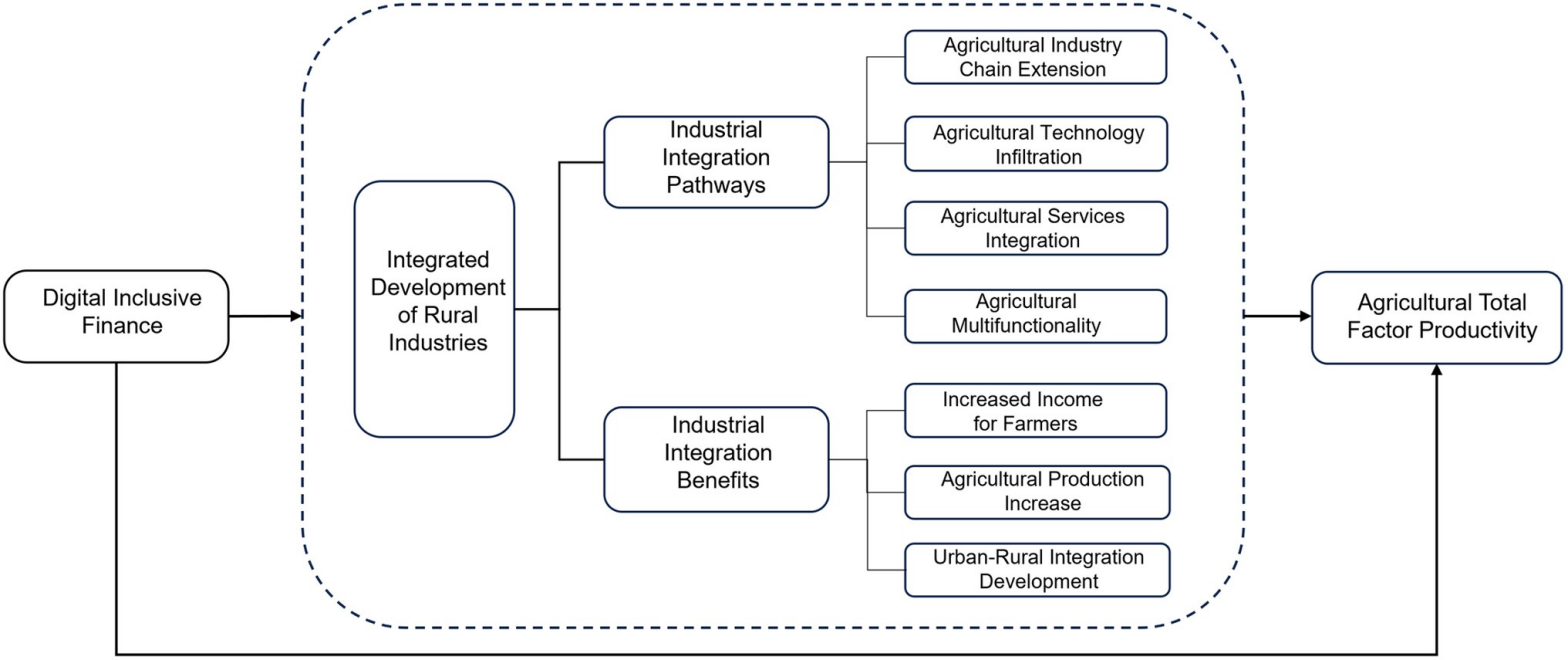
The influence of digital inclusive finance on agricultural total factor productivity.

Drawing from the aforementioned theoretical analysis, this study establishes a model (refer to Fig 1) delineating the influence of digital inclusive finance on agricultural total factor productivity. This model discerns two primary facets: firstly, the direct impact of digital inclusive finance on agricultural production efficiency; secondly, the intermediary role played by digital inclusive finance in affecting agricultural total factor productivity through the development of rural industry integration.

## Research design

### Data source

This paper reports the results of an analysis of data from 11 prefecture-level cities in Zhejiang Province, China, from 2011 to 2020. The data used in this analysis consist of (i) digital inclusive finance data from prefecture-level cities and (ii) statistical data from prefecture-level cities in Zhejiang Province. The relevant indicators of digital inclusive finance were sourced from the digital inclusive finance database developed by Guo et al. (2020) [40]. Data related to agricultural inputs and outputs, control variables, and intermediary variables were collected from various editions of the Zhejiang Statistical Yearbook, the China City Statistical Yearbook, and the China Statistical Yearbook. Linear interpolation filled in the gaps when data were missing.

### Variable selection

#### Dependent variable

The dependent variable in this study was agricultural TFP, which was calculated using agricultural input and output data from prefecture-level cities in Zhejiang Province. For the output variable, the literature usually uses various indicators such as the total value of agriculture, forestry, animal husbandry, and fishery output, the total value of primary-industry output, and the value added by agriculture. This study followed the approach of Chaojing et al. (2018) [41] and Guanglin et al. (2023) [42], who used the broad measure of the total value of agriculture, forestry, animal husbandry, and fishery output (in billions of RMB) as the output variable, adjusted for constant (2010) prices.

The input variables were taken from the literature [20, 43, 44]. We selected the three most common factors for calculating China’s agricultural TFP: labor, land, and machinery. Specifically, labor input is the number of employees engaged in agriculture, forestry, animal husbandry, and fishery (in tens of thousands). Land input is the total area of crops sown (in thousands of hectares). Finally, machinery input is the total power of agricultural machinery (in tens of thousands of kilowatts).

Due to the flexibility and non-necessity to specify production functions, the DEA-Malmquist index method was chosen in this study to calculate agricultural TFP for the 11 prefecture-level cities in Zhejiang Province. The objective of the DEA-Malmquist index method is to construct a production frontier envelopment surface by observing actual production data points, mapping non-DEA-efficient decision-making units to the DEA-efficient production frontier, and subsequently evaluating the efficiency of each decision-making unit by comparing its relative distance from the DEA-efficient production frontier. Assuming a constant return to scale *C* and a strong disposability *S* of input, the possible production set for each decision-making unit in period *t* is

$$\begin{array}{c} P^{t}\left(x^{t}|C,S\right)=\{\begin{array}{c} \left(y_{i}^{t},\cdots ,y_{M}^{t}\right)\\ \sum_{k=1}^{K}z_{k}^{t}y_{km}^{t}\geq y_{km}^{t},m=1,\cdots ,M\\ \sum_{k=1}^{K}z_{k}^{t}x_{kn}^{t}\leq x_{kn}^{t},n=1,\cdots ,N\\ z_{k}^{t}\geq 0,n=1,\cdots ,N \end{array}\} \end{array}$$ (1)

where *z* is a density variable that reflects the weights assigned to individual decision units when evaluating technical efficiency.

Building upon this foundation and using further advancements by Caves et al. (1982) and Färe et al. (1992), the DEA is combined with the Malmquist index, making it the most commonly used analytical method in the field [45, 46]. Considering variations over time, such as from period *t* to *t* + 1, the input-oriented Malmquist productivity index is

$$\begin{array}{c} M_{i,t+1}\left(x_{i}^{t},y_{i}^{t},x_{i}^{t+1},y_{i}^{t+1}\right)=\underset{TEC_{i}^{t+1}}{\underset{︸}{\frac{D_{i}^{t+1}\left(x_{i}^{t+1},y_{i}^{t+1}|C\right)}{D_{i}^{t}\left(x_{i}^{t},y_{i}^{t}|C\right)}}}\cdot \underset{TP_{i}^{t+1}}{\underset{︸}{\left[\frac{D_{i}^{t}\left(x_{i}^{t+1},y_{i}^{t+1}|C\right)}{D_{i}^{t+1}\left(x_{i}^{t+1},y_{i}^{t+1}|C\right)}\cdot \frac{D_{i}^{t}\left(x_{i}^{t},y_{i}^{t}|C\right)}{D_{i}^{t+1}\left(x_{i}^{t},y_{i}^{t}|C\right)}\right]}} \end{array}$$ (2)

where the first factor on the right-hand side is the technical efficiency change from period *t* to *t* + 1 for the decision-making unit. The second factor is the change in technical progress from period *t* to *t* + 1 for the decision-making unit.

For variable returns to scale, Färe et al. (1994) [47] argued that technical efficiency be further decomposed into the pure technical efficiency index and the scale efficiency index:

$$\begin{array}{c} \begin{array}{cc} TEC_{i}^{t+1} & =\frac{D_{i}^{t+1}\left(x_{i}^{t+1},y_{i}^{t+1}|C\right)}{D_{i}^{t}\left(x_{i}^{t},y_{i}^{t}|C\right)}=\frac{D_{i}^{t+1}\left(x_{i}^{t+1},y_{i}^{t+1}|C\right)/D_{i}^{t+1}\left(x_{i}^{t+1},y_{i}^{t+1}|V\right)}{D_{i}^{t}\left(x_{i}^{t},y_{i}^{t}|C\right)/D_{i}^{t}\left(x_{i}^{t},y_{i}^{t}|V\right)}\\ & \cdot \frac{D_{i}^{t+1}\left(x_{i}^{t+1},y_{i}^{t+1}|V\right)}{D_{i}^{t}\left(x_{i}^{t},y_{i}^{t}|V\right)}=\underset{SEC_{i}^{t+1}}{\underset{︸}{\frac{SE_{i}^{t+1}\left(x_{i}^{t+1},y_{i}^{t+1}\right)}{SE_{i}^{t}\left(x_{i}^{t},y_{i}^{t}\right)}}}\cdot \underset{PEC_{i}^{t+1}}{\underset{︸}{\frac{D_{i}^{t+1}\left(x_{i}^{t+1},y_{i}^{t+1}|V\right)}{D_{i}^{t}\left(x_{i}^{t},y_{i}^{t}|V\right)}}} \end{array} \end{array}$$ (3)

Building upon Eq (2), the Malmquist index (TFP index) is expressed as

$$\begin{array}{c} \begin{array}{cc} TFP_{i}^{t+1} & =M_{i,t+1}\left(x_{i}^{t},y_{i}^{t},x_{i}^{t+1},y_{i}^{t+1}\right)\\ & =TEC_{i}^{t+1}\times TP_{i}^{t+1}=SEC_{i}^{t+1}\times PEC_{i}^{t+1}\times TP_{i}^{t+1} \end{array} \end{array}$$ (4)

Eq (4) provides the methodological foundation for exploring the evolution of regional agricultural TFP.

#### Core explanatory variables

The core explanatory variable in this study is the digital inclusive finance index. We collected sample data from 11 prefecture-level cities in Zhejiang province. This index encompasses data at three levels: provincial, municipal, and county. It was computed by the Peking University Digital Finance Research Center and is based on digital financial service data provided by Ant Financial. The digital inclusive finance index scores were calculated by using dimensionless methods and an analytic hierarchy process. This approach used the digital inclusive finance index system [40].

#### Mediating variable

The mediating variable in this paper is the level of rural industrial integration. Building upon existing research [33, 48, 49], this paper combines principles of scientific indexing and data availability to construct indicators from both the pathways and benefits of industrial integration. Furthermore, considering the content and objectives of industrial integration development, the pathways are categorized into four secondary indicators and the benefits of industrial integration are divided into three secondary indicators. Subsequently, nine tertiary indicators are identified based on the secondary indicators. These collectively form the basis for constructing a comprehensive rural industrial integration index to evaluate the level of rural industrial integration. See Table 1 for the specific index system.

(1) *Industrial Integration Pathways*. Rural industrial integration aims to break the boundaries between the primary sectors and the secondary or tertiary sectors in rural areas. It seeks to continuously expand agricultural production, improve living standards, and enhance ecological functionality through various pathways, such as the extension of agricultural-industry chains, the penetration of agricultural technology, the integration of agricultural services, and the promotion of agricultural multifunctionality. This approach is designed to be synergistic (1 + 1 + 1 > 3). The industrial integration pathways comprise four secondary indicators:
(a) *Agricultural Industry Chain Extension*. This pathway focuses on developing the processing and sales of agricultural products to enhance their added value (i.e., the complete closure of the industrial chain by extending the industrial chain based on traditional agricultural production models, from agricultural product production, to agricultural product processing, and to agricultural product sales).
(b) *Agricultural Technology Infiltration*. This pathway introduces agricultural machinery and information technologies such as the Internet of Things and big data. This accelerates the development of smart agriculture and rural e-commerce, improving cross-sector integration and increasing agricultural production efficiency.
(c) *Agricultural Services Integration*. This pathway signifies the interaction between agriculture and services within the agricultural industry chain, such as agricultural input distribution, agricultural technology extension, and marketing of agricultural products.
(d) *Agricultural Multifunctionality*. This pathway refers to the continuous expansion of agriculture’s social, economic, cultural, and ecological functions. It leverages green ecological resources to promote diversified development in sectors such as culture, tourism, and wellness, ultimately integrating the primary sectors with the secondary and tertiary sectors.
(2) *Industrial Integration Benefits*. Promoting increased income for farmers, higher agricultural production, and reducing the urban-rural income gap are the primary objectives of rural industrial integration. These objectives also serve as specific indicators of the benefits of industrial integration. The industrial integration benefits encompass the following three secondary indicators:
(a) *Increased income for farmers*. This indicator aims to diversify and stabilize farmer income through industrial integration, opening up additional employment opportunities for rural residents.
(b) *Agricultural production increase*. This indicator expands the agricultural industry and improves resource efficiency through advanced technologies, leading to higher crop yields per unit of land.
(c) *Urban-rural integration development*. This indicator promotes the flow of resources between urban and rural areas to drive rural industrial integration and, ultimately, to reduce the urban-rural income gap.

**Table 1:** Evaluation index for integration of rural industries.

Target layer	First indicators	Second indicators	Third indicators	Indicator description	Attribute
Integrated development of rural industries (100%)	Industrial integration pathways (61.59%)	Agricultural industry chain extension (8.86%)	Industrial structure (8.86%)	Fraction of added value of the secondary and tertiary industries with respect to total GDP	Positive
		Agricultural technology infiltration (9.98%)	Degree of agricultural mechanization (9.42%)	Ration of total power of agricultural machinery to total arable land area (kW/ha)	Positive
			Internet development (10.55%)	Ration of number of international Internet users to total number of permanent residents	Positive
		Agricultural services integration (9.98%)	Growth rate of per capita output value (9.98%)	Annual average cycle-increase rate of the ratio of agricultural, forestry, animal husbandry, and fishing professional and auxiliary activity output value to rural population (10000 yuan/person)	Positive
		Agricultural multifunctionality (22.78%)	Fraction of employees in secondary and tertiary industries (11.11%)	1-fraction of employees in primary industry	Positive
			Degree of rural ecological protection (11.67%)	The ratio of intensity of pesticide use to planting area (tons per thousand hectares)	Negative
	Industrial integration benefits (38.41%)	Increased income for farmers (12.24%)	Growth rate of per capita disposable income of farmers (12.24%)	Annual average cycle-increase rate of per capita disposable income of farmers	Positive
		Agricultural production increase (12.80%)	Grain yield per hectare (12.80%)	Grain sown per hectare (kg)	Positive
		Urban-Rural integration development (13.37%)	Per capita income ratio of urban and rural residents (13.37%)	Ratio of per capita income of urban residents to per capita income of rural residents	Negative

To quantitatively assess the level of rural industrial integration development, this paper calculates the comprehensive index of rural industrial integration development using the entropy method based on the indicator system presented in Table 1. The calculation process involves the following steps:

First, the indicator data are standardized as follows:

For positively oriented indicators,

$$\begin{array}{c} y_{ij}=\frac{\left(x_{ij}-\text{min}\;x_{j}\right)}{\left(\text{max}\;x_{j}-\text{min}\;x_{j}\right)}+0.0001 \end{array}$$ (5)

For inversely oriented indicators,

$$\begin{array}{c} y_{ij}=\frac{\left(\text{max}\;x_{j}-x_{ij}\right)}{\left(\text{max}\;x_{j}-\text{min}\;x_{j}\right)}+0.0001 \end{array}$$ (6)

Here, *x*_*i*_*j* is the observed value of indicator *j* for the prefecture-level city *i*, while max *x*_*j*_ and min *x*_*j*_ denote the maximum and minimum observed values, respectively, for indicator *j*.

Next, the weights for each indicator are determined as follows [49]:

(1) *Contribution calculation*. The contribution of prefecture-level city *i* to indicator *j* is $p_{ij}=\frac{y_{ij}}{\sum_{i}y_{ij}}$.
(2) *Entropy calculation*. The entropy for indicator *j* is $e_{j}=-\frac{1}{\text{ln}\;n}\sum_{i}p_{ij}\;\text{ln}\;p_{ij}$, where n is the total number of prefecture-level cities.
(3) *Redundancy calculation*. The redundancy for prefecture-level city *i* is *g*_*i*_ = 1 − *e*_*j*_.
(4) *Weight assignment*. Finally, the weight *w*_*i*_ for each indicator is *w*_*i*_ = *g*_*j*_/∑_*j*_ *g*_*j*_.

Ultimately, the comprehensive index of rural industrial integration for prefecture-level city *i* is

$$\begin{array}{c} f_{i}=\sum_{j}w_{j}y_{ij} \end{array}$$ (7)

This process produces an index that comprehensively assesses the level of rural industrial integration by considering the various indicators and their respective weights, ensuring a rigorous and systematic evaluation.

#### Control variables

We now examine how digital inclusive finance affects agricultural TFP at the level of prefecture cities. The selection of control variables is influenced by the methods used in the studies of Tang et al. (2022) [44] and Zheng and Li (2022) [20]. The following eight control variables are used for this analysis: the fraction of technology research and development investment, per capita grain planting area, human capital quality, informatization, financial development, degree of openness to foreign trade, and rural electricity consumption. Table 2 presents the variable assignments and descriptive statistics.

**Table 2:** Variable assignment and descriptive statistics.

Variable	Variable assignment	Mean value	Standard deviation
Agricultural total factor productivity	Refer to Eq (4) for details	1.0663	0.0849
Digital inclusive finance index	Original value of digital inclusive finance index/100	2.0805	0.69564
Integrated development of rural industries index	Refer to Eq (7) for details	0.5068	0.0728
Fraction of technology research and development investment	Total social research and experimental development (R&D) expenditure/regional GDP (100 million yuan/100 million yuan)	0.0264	0.0094
Per capita grain planting area	Grain planting area/year-end permanent population (1000 hectares/10000 people)	0.2411	0.1416
Human capital	Number of students in regular high schools/ Year-end permanent population (person/person)	0.0420	0.0078
Informatization	Number of fixed-telephone users/year-end permanent population (person/person)	0.2528	0.0831
Financial development	Year-end deposit balance of financial institutions/year-end permanent population (10000 yuan/person)	9.5859	4.6371
Degree of openness	Total import and export volume/regional GDP (100 million yuan/100 million yuan)	0.4453	0.2443
Rural electricity consumption	Rural electricity consumption/rural population (100 million kilowatt hours/10000 people)	0.4691	0.3350

### Model construction

This paper primarily establishes a two-way fixed effects model to examine how digital inclusive finance affects agricultural TFP. The choice of this model is motivated by several factors:

(1) *Bidirectional causality*. Bidirectional causality may exist between digital inclusive finance and productivity growth. A econometric model based on panel data is deemed suitable to mitigate endogeneity issues [44].
(2) *Multifaceted influences*. Agricultural TFP is affected by numerous factors, including both observable input and output elements, as well as unobservable factors such as the willingness to adopt new agricultural technologies. Adopting a two-way fixed effects model can mitigate how unobservable factors affect agricultural TFP, thereby enhancing the model’s estimation accuracy [50].

### Benchmark regression model

To examine how digital inclusive finance affects agricultural TFP, this paper constructs, in conjunction with research hypothesis 1(H1), the following panel econometric model:

$$\begin{array}{c} \text{ln}\;TFP_{it}=\alpha_{0}+\alpha_{l}\;\text{ln}\;DIF_{it}+\alpha_{2}\;\text{ln}\;CONTROL_{it}+\mu_{i}+\theta_{t}+\varepsilon_{it} \end{array}$$ (8)

where subscripts *i* and *t* refer to prefecture-level city *i* and year *t*, and where TFP is the dependent variable representing agricultural TFP calculated by using the DEA-Malmquist index. DIF is the digital inclusive finance index, and CONTROL contains the control variables. *α*_0_ is the intercept, *α*_1_ and *α*_2_ are the estimated coefficients, *μ*_*i*_ and *θ*_*t*_ are space-fixed effects and time-fixed effects, respectively, and *ε*_*it*_ is the random disturbance term.

### Mediating effect model

In conjunction with research hypothesis 2(H2), we now investigate how digital inclusive finance enhances agricultural TFP. This paper uses a mediating effect model. Considering the inherent endogeneity issues in traditional three-stage mediating effect models and following the operational recommendations proposed by Jiang (2022) [51] for analyzing mediating effects, this paper only assesses how digital inclusive finance affects the mediating variables in the empirical part. Therefore, building upon model 8, this paper establishes the following model of the mediating effects:

$$\begin{array}{c} \text{ln}\;M_{it}=\gamma_{0}+\gamma_{l}\;\text{ln}\;DIF_{it}+\gamma_{2}\;\text{ln}\;CONTROL_{it}+\mu_{i}+\theta_{t}+\varepsilon_{it} \end{array}$$ (9)

where *M* is the comprehensive index of rural industrial integration, signifying the variable that mediates how digital inclusive finance affects agricultural TFP, *γ*_0_ is the intercept, and *γ*_1_ and *γ*_2_ are the estimated coefficients. The other variables retain the same meaning as above.

## Impact of digital inclusive finance on agricultural total factor productivity

### Benchmark regression test

Drawing on the practices used by Zheng and Li (2022) [20] and Shen et al. (2023) [25], this study uses a fixed-effects model to analyze how digital inclusive finance affects agricultural TFP. The analysis considers four models: (1) a single-time fixed-effects model, (2) a single-area fixed-effects model, (3) a two-way fixed effects model without control variables, and (4) a two-way fixed effects model with control variables. The calculated results are shown in Table 3. It can be found that with model (1), the digital inclusive finance index is not statistically significant. With model (2), the digital inclusive finance index is significant at the 10% level but suppresses agricultural TFP. With model (3), the digital inclusive finance index is significant at the 10% level. With model (4), digital inclusive finance significantly affects agricultural TFP at the 1% level. Specifically, a single-unit increase in digital inclusive finance development leads to an average increase of 0.3737 units in regional agricultural TFP. Unlike the single fixed-effects model, the two-way fixed effects model includes two control variables, time and individual, which mitigates the issues related to omitted variables, thereby increasing the significance and stability of the model’s output. Furthermore, the inclusion of control variables elevates the significance of digital inclusive finance for agricultural TFP. As a result, this study adopts the two-way fixed effects model with control variables [model (4)] to analyze how digital inclusive finance affects agricultural TFP and uses cluster-robust standard errors to address heteroscedasticity issues.

**Table 3:** Variable assignment and descriptive statistics.

Variables all indicators are logarithms	Explained variable: logarithm of agricultural total factor productivity			
	Model (1)	Model (2)	Model (3)	Model (4)
Digital inclusive finance index	0.3737 (0.4244)	−0.1027* (0.0593)	0.3722* (0.1995)	0.3737*** (0.1009)
Proportion of technology research and development investment	0.0616 (0.0838)	0.0802 (0.0775)		0.0616* (0.0309)
Per capita grain-planting area	−0.2166** (0.1088)	−0.0422 (0.0715)		−0.2166* (0.1031)
Human capital quality	0.0913 (0.1865)	−0.0530 (0.1567)		0.0913 (0.0688)
Informatization	0.0529 (0.1168)	0.0190 (0.0744)		0.0529 (0.0967)
Financial development	0.0718 (0.1663)	0.1343 (0.1293)		0.0718 (0.0985)
Degree of openness to foreign trade	−0.0293 (0.0622)	0.0200 (0.0580)		−0.0293 (0.0232)
Rural electricity consumption	0.0245 (0.1124)	−0.0560 (0.1011)		0.0245 (0.0816)
Constant	0.3524 (0.8077)	−0.1160 (0.6845)	0.1648*** (0.0497)	0.3524 (0.3065)
N	110	110	110	110
*R* ^2^	0.2046	0.0607	0.1524	0.2046
adj. *R*^2^	−0.0573	−0.1251	0.0667	0.0577
Control variables	Yes	Yes	No	Yes
Year fixed	Yes	No	Yes	Yes
Region fixed	No	Yes	Yes	Yes

Note:

***, **, and * represent significance levels of 1%, 5%, and 10%, respectively, with the estimated standard error in parentheses.

The coefficient for the core explanatory variable, the digital inclusive finance index, is positive with a p value below 0.01, signifying that digital inclusive finance has a significant positive effect on Zhejiang’s agricultural TFP. This finding is consistent with the conclusion of Zheng and Li (2022) [20]. With the rapid advancement of digital technology, financial services can innovate through technology-enabled approaches and reach a greater portion of agricultural, rural, and farming populations, thereby significantly enhancing agricultural TFP.

For the control variables, the coefficient for the fraction of research and development investment in technology is positive and significant at the 10% level. A one-unit increase in the fraction of research and development investment in technology leads to an average increase of 0.0616 units in regional agricultural TFP. Yang et al. (2019) [52] found that increased investment in agricultural technology research and development significantly promotes agricultural production. This suggests that increased research and development investment in technology enhances technological capabilities, thereby improving resource-utilization efficiency and reducing the cost of agricultural production.

The coefficient for per capita grain-planting area is negative and significant at the 10% level, indicating that per capita arable land correlates negatively with agricultural TFP. This result is consistent with those of Yao et al. (2022), who observed that economically developed grain-producing regions, including Zhejiang, experienced a significant reduction in the planting area of grain crops due to rapid urbanization and the development of leisure agriculture [53]. This phenomenon negatively impacted grain production due to the reduction in scale. As agricultural technology progresses and labor productivity increases, agricultural production efficiency must continuously improve to compensate for the challenges posed by the decreasing per capita arable land on grain yield and quality.

The coefficient for human capital quality is positive. Each one-unit increase in human capital quality corresponds to an average increase of 0.0913 units in regional agricultural TFP. According to Ma and Qu (2021) [54], an increase in human capital quality improves the skills of low-income groups such as farmers, transforms their attitudes and ways of thinking, enhances work efficiency, and promotes high-quality development in agriculture and rural economies.

The coefficient for informatization is positive. Each one-unit increase in informatization corresponds to an average increase of 0.529 units in regional agricultural TFP. Agricultural informatization optimizes resource allocation in agriculture, reduces the cost of information acquisition for agricultural products, more effectively guides the production and circulation of agricultural products, and enhances production efficiency, management, and operational decision-making in agriculture. It accelerates technological progress in agriculture, making it a key factor driving the growth of agricultural TFP [55].

The coefficient for financial development is positive. Each unit increase in financial development increases agricultural TFP by 0.0718 units. Improving the level of financial development further improves the rural financial market, facilitating diversified savings options and financing channels for rural economic entities. Financial institutions encourage savings to be converted into investments, promoting capital accumulation for agricultural development, which in turn drives rural economic growth [56].

The coefficient for the degree of openness is negative. For each unit increase in the degree of openness, agricultural TFP decreases by 0.0293 units. Although an openness foreign trade transforms the local agricultural environment and expands China’s agricultural trade market, it also exposes China’s domestic agricultural markets to fierce global competition. Issues such as the inability of traditional agricultural products to establish a presence in international markets, significant pressure from the World Trade Organization tariff rules, and low prices discouraging farmers from active cultivation have led to challenges, including food security problems [57]. Consequently, agricultural development faces growing competitive pressure, which hinders the development of the agricultural economy.

The coefficient for rural electricity consumption is positive. Each one-unit increase in rural electricity consumption corresponds to an average increase of 0.0245 units in regional agricultural TFP. An increase in rural electricity consumption promotes mechanization in agriculture, reducing labor intensity and significantly enhancing agricultural production efficiency. Additionally, support from electric utilities drives the application of new technologies and methods in agriculture, improving agricultural science and technology, which ultimately boosts agricultural TFP.

### Endogeneity discussion and robustness tests

#### Endogeneity discussion

From an empirical analysis perspective, the benchmark regression model may suffer from endogeneity issues. Digital inclusive finance and agricultural TFP might simultaneously be influenced by a range of unobservable factors, such as farmers’ individual capabilities, agricultural development perspectives, and future expectations. This situation could bias the regression coefficients for digital inclusive finance. To mitigate potential endogeneity problems arising from these factors, we follow the approach of Yi and Zhou (2018) [58] by constructing a “Bartik instrument,” which involves the product of a one-period lagged digital inclusive finance index and the first-order differential digital inclusive finance index.

Table 4 shows the estimation of instrumental variables using a two-stage least squares method. The first-stage instrumental variable coefficient demonstrates statistically significant results different from zero, and a weak instrument test (F-statistic of 17.27, exceeding 10) suggests a relatively low likelihood of weak instrument issues. The second-stage regression results reveal that digital inclusive finance is significant at the 5% level. Thus, with endogeneity taken into account, digital inclusive finance continues to significantly promote agricultural TFP, further underscoring the robustness and reliability of the regression model in this study.

**Table 4:** Regression results for instrumental variable.

Variables	Dependent variable of first stage	Dependent variable of second stage
	Logarithm of digital inclusive finance index	Logarithm of agricultural total factor productivity
Instrumental variable (Digital inclusive finance index lag first order Digital inclusive finance index first order difference)	0.2030*** (0.0495)	
Logarithmic value of digital inclusive finance index		0.7821** (0.3884)
Constant	0.7338*** (0.2054)	−0.1481 (0.4234)
N	99	99
*R* ^2^	0.9951	0.1825
Control variables	Yes	Yes
Year fixed	Yes	Yes
Region fixed	Yes	Yes

Note:

***, **, and * represent significance levels of 1%, 5%, and 10%, respectively, with robust standard errors in parentheses. The results include control variables and are controlled for the fixed effects of region and year.

#### Robustness tests

This study uses two methods to ensure the robustness of the empirical analysis results. First, a different treatment for the core explanatory variable is considered. With the baseline regression model describing how digital inclusive finance affects agricultural TFP, the original values of digital inclusive finance are divided by 100, and then the natural logarithm is taken. This approach tests how the original values of digital inclusive finance affect agricultural TFP (see results in Column 1 of Table 5). The results indicate that the original values of digital inclusive finance have a significant positive effect on agricultural TFP, which is consistent with the baseline regression results, confirming the robustness of the estimates of how digital inclusive finance affects agricultural TFP.

**Table 5:** Regression results for robustness test.

Variables	(1) Replace core explanatory variables	(2) Winsorize
Original value of digital inclusive finance index	0.0032* (0.0018)	
Logarithm of digital inclusive finance index		0.3489** (0.1113)
Constant	0.1804 (0.2313)	0.2615 (0.3406)
N	110	110
*R* ^2^	0.2122	0.2042
adj. *R*^2^	0.0666	0.0571
Control variables	Yes	Yes
Year fixed	Yes	Yes
Region fixed	Yes	Yes

Note:

***, **, and * represent significance levels of 1%, 5%, and 10%, respectively, with robust standard errors in parentheses. The results included control variables and are controlled for the fixed effects of city and year.

Second, trimming is applied. Outliers are removed from all indicators in the baseline regression model, and the top and bottom 1% of the data are trimmed (i.e., eliminated). As shown in Column 2 of Table 5, digital inclusive finance significantly promotes agricultural TFP at the 5% level, further confirming the robustness of the baseline regression results.

### Mediating effect test

The results in Table 6 show that the regression coefficient between digital inclusive finance and the rural industry integration development composite index is 0.9844, which passes the 5% significance test. The results indicate that digital inclusive finance enhances agricultural TFP by promoting the integration of rural industry, thus validating research hypothesis 2(H2).

**Table 6:** Results of mediating effect test.

Variables	(1) Logarithm of agricultural total factor productivity	(2) Logarithm of integrated development of rural industries index
Logarithm of digital inclusive finance index	0.3737*** (0.1009)	0.9844** (0.3768)
Constant	−3.3439*** (0.5509)	−8.4475** (2.7668)
N	110	110
*R* ^2^	0.2129	0.7520
adj. *R*^2^	0.0572	0.7029
Control variables	Yes	Yes
Year fixed	Yes	Yes
Region fixed	Yes	Yes

Note:

***, **, and * represent significance levels of 1%, 5%, and 10%, respectively, with robust standard errors in parentheses. The results include control variables and are controlled for the fixed effects of city and year.

In the context of promoting comprehensive rural revitalization and building a strong agricultural nation, the focus of rural industry integration development in Zhejiang has shifted from ordinary farmers to new agricultural operators such as family farms, rural professional cooperatives, leading agricultural enterprises, and rural collective economic organizations. The scale and demand for rural industry integration have grown, and funding is increasingly needed. Traditional financial institutions are often reluctant to provide financial services in rural areas due to the seasonality and dispersion of agricultural-production funding, as well as the challenging task of evaluating and quantifying individual creditworthiness.

Digital inclusive finance, as a new format combining modern digital technology with traditional inclusive finance, offers a solution to alleviate farmers’ credit constraints and the issue of asymmetric loan information. Leveraging information technologies such as big data, cloud computing, and artificial intelligence, digital inclusive finance can expand the scope and accessibility of financial services, making full use of its role to enhance the efficiency and reach of information [25]. Digital inclusive finance can mitigate corporate financing constraints, thereby boosting innovation among businesses [59]. In turn, this approach promotes innovation in agriculture, such as smart farming, biotechnology, and ecological agriculture, facilitating cross-sector penetration of agricultural technology and advancing rural industry integration [48].

Digital inclusive finance can reduce financing costs and thresholds for farmers, offering financial support for agricultural operators and allowing them to participate in the agricultural machinery service market. This strategy drives the development of the agricultural service industry [60]. The development of digital inclusive finance widens the borrowing channels for returnees engaged in entrepreneurial activities in rural areas, encouraging farmers to leverage local resources for entrepreneurial opportunities, which increases job opportunities and incomes [61]. Moreover, digital inclusive finance further promotes agriculture, ecology, and tourism. In the context of ecological functions, digital inclusive finance integrates with the goals of ecological agriculture development, constructing a green financial system, reinforcing environmental regulation for green development, and achieving the green transformation of agriculture and capital [62]. In the realm of tourism, digital payments cater to urban residents’ digital payment habits, enhancing rural leisure tourism for both urban and rural residents and thus promoting rural tourism [33].

Finally, digital inclusive finance provides rural residents with a new channel to comprehensively, accurately, and promptly access financial knowledge. This strategy, in turn, enhances their financial literacy and improves their ability to collect and analyze market information, enabling them to identify entrepreneurial opportunities and make family decisions that align with their interests. The fairness, efficiency, affordability, and penetration of digital inclusive finance boost economic growth and industrial development, making it a key element in promoting the transformation and upgrade of rural industries and enhancing agricultural quality and efficiency [35].

### Heterogeneity analysis

The essence of digital inclusive finance remains rooted in the financial domain, and its development is intricately linked to factors such as local socio-economic conditions, financial deepening, population density, and infrastructure development [63]. The regional disparities arising from these factors result in significant regional heterogeneity in the level of digital inclusive finance in rural areas [64], which leads to regional heterogeneity in how digital inclusive finance affects agricultural development and by which mechanisms.

The inclusive nature of digital inclusive finance enables it to mitigate financial exclusion while providing financial services to both agriculturally advanced and underdeveloped regions. In contrast with agriculturally advanced regions, underdeveloped regions lack sufficient capital resources. The development of digital inclusive finance offers a potential solution to provide financial services in these underdeveloped agricultural regions [65] and reduces credit constraints. Given that capital elements are relatively scarce in these regions, they tend to emphasize developing digital inclusive finance. Therefore, the influence of digital inclusive finance on agricultural development can vary due to different levels of regional agricultural development.

#### Regional heterogeneity

The prefecture-level cities in Zhejiang differ in their regional geographical characteristics, which could lead to variations in agricultural production models and potentially influence agricultural TFP. Based on historical data from the “Zhejiang Statistical Yearbook,” Zhejiang is divided into two main regions: Northeast Zhejiang and Southwest Zhejiang, and a regression analysis was conducted separately for these regions. Northeast Zhejiang includes Hangzhou, Ningbo, Jiaxing, Huzhou, Shaoxing, and Zhoushan, encompassing all counties (cities and districts). Southwest Zhejiang comprises Wenzhou, Jinhua, Quzhou, Taizhou, and Lishui, encompassing all counties (cities and districts).

As shown in Table 7, the impact of digital inclusive finance on agricultural TFP in Northeast Zhejiang is greater than its impact in Southwest Zhejiang, which can be explained by two possible reasons: First, arable land in Northeast Zhejiang, traditionally known as the “land of fish and rice,” consists of relatively flat terrain, providing favorable conditions for agricultural development [66]. Second, Northeast Zhejiang is adjacent to Shanghai, which facilitates trade and transportation and results in higher levels of economic development and infrastructure. Additionally, by leveraging its geographic proximity to Hangzhou, Northeast Zhejiang has an advantage in the development of digital inclusive finance. Thus, the impact of digital inclusive finance on agricultural TFP in Northeast Zhejiang is more pronounced than in Southwest Zhejiang.

**Table 7:** Results of heterogeneity test.

Variables	Region		Agricultural development level		
	Northeast Zhejiang	Southwest Zhejiang	First echelon	Second echelon	Third echelon
Logarithm of digital inclusive finance index	0.5801 (0.3125)	0.3218 (0.5808)	1.2825 (1.0186)	3.2785** (0.7436)	0.1292 (0.3628)
Constant	0.6040 (1.3949)	−0.6100 (0.9786)	1.6882 (3.4306)	5.1978** (1.4763)	1.9044 (1.1186)
N	60	50	30	40	40
*R* ^2^	0.2398	0.5846	0.5727	0.6268	0.4240
adj. *R*^2^	-0.0940	0.3434	-0.1266	0.3070	-0.0697
Control variables	Yes	Yes	Yes	Yes	Yes
Year fixed	Yes	Yes	Yes	Yes	Yes
Region fixed	Yes	Yes	Yes	Yes	Yes

Note:

***, **, and * represent significance levels of 1%, 5%, and 10%, respectively, with robust standard errors in parentheses. The results include control variables and are controlled for fixed effects of city and year.

#### Heterogeneity in agricultural development levels

Since the 18th Party Congress, Zhejiang has significantly emphasized rural development and aimed to create a comprehensive agricultural region where agriculture, forestry, animal husbandry, and fishery industries can develop harmoniously. The cities in Zhejiang have made remarkable progress in optimizing the industrial structure of agriculture, forestry, animal husbandry, and fisheries. To investigate how digital inclusive finance affects agricultural TFP under varying levels of agricultural development, three strata are formed by ranking the cities according to their levels of agricultural development, which is based on the average total output value of agriculture, forestry, animal husbandry, and fisheries over the past three years. The first stratum includes Ningbo, Taizhou, and Hangzhou; the second stratum Shaoxing, Zhoushan, Wenzhou, and Jinhua; and the third stratum Huzhou, Jiaxing, Lishui, and Quzhou.

As indicated in Table 7, digital inclusive finance on agricultural TFP has the greatest impact on the second stratum, with a positive and statistically significant coefficient at the 5% significance level. The first stratum experiences a slightly lower impact, and the third stratum experiences the least impact. Analyzing the results from the second and third strata reveals that, as the total output value of agriculture, forestry, animal husbandry, and fisheries increases, the impact of digital inclusive finance on agricultural TFP increases. However, the results from the first and second strata suggest that when agricultural development increases past a certain threshold, the magnitude of the impact of digital inclusive finance on agricultural TFP tends to decrease. This observation has several possible explanations: First, developing digital inclusive finance can improve the capital supply in agriculturally advanced regions. However, because these regions already possess relatively abundant capital, the impact of digital inclusive finance is limited [10]. Second, first-stratum cities have high levels of economic development, and financial resources already largely satisfy the demands of agricultural production. The development of digital inclusive finance may thus expand the financing channels but has a relatively minor impact [21]. In contrast, second-stratum cities have relatively underdeveloped rural financial sectors, making the impact of digital inclusive finance more pronounced. In third-stratum cities, the level of digital inclusive finance remains insufficient to fully satisfy the requirements of agricultural development, resulting in a less significant impact of digital inclusive finance on agricultural TFP.

## Conclusion and recommendations

Based on panel data spanning from 2011 to 2020 and across 11 prefecture-level cities in Zhejiang, this paper assesses agricultural TFP in these regions, constructs a rural industry integration development index, and uses a two-way fixed-effects model to analyze how digital inclusive finance affects agricultural TFP. The analysis also explores mediating mechanisms and effects of heterogeneity. The results lead to the following conclusions:

First, digital inclusive finance significantly improves agricultural TFP in Zhejiang.

Second, rural industry integration plays a positive mediating role in enhancing agricultural TFP driven by digital inclusive finance.

Third, regional heterogeneities and heterogeneities based on the level of agricultural development vary the influence of digital inclusive finance on agricultural TFP. The impact of digital inclusive finance on agricultural TFP is more pronounced in Northeast Zhejiang, especially in second-tier areas.

Based on these research findings, the following policy recommendations are proposed:

First, a comprehensive elevation of rural digital inclusive financial services is imperative to reinforce the profound integration of digital finance with agricultural development. Rural enterprises can leverage the advancements in digital technology to enhance internal risk control and management, reduce operational and innovative costs, and elevate the overall productivity of agricultural factors. Farmers should actively engage in acquiring new knowledge and skills, enhancing their digital literacy to adapt to the digitalized financial service models. By aligning with the diverse developmental needs of rural industries, this will ignite innovative entrepreneurial capabilities, thereby propelling the digitalization and intelligent evolution of agriculture, ultimately facilitating the revitalization of integrated rural industries.

Second, creating an integrated financial ecosystem for rural industries is essential to fully harness the synergistic effects of urban-rural integration in augmenting the productivity of all agricultural factors. Financial institutions should consider digital technology as an indispensable tool for fostering agricultural development. This involves developing intelligent identification tools, enhancing the efficiency and quality of agricultural-related credit operations, and employing big data technology to upgrade risk control methodologies. This approach will enable effective supervision of fund usage, leading to a reduction in default rates and fostering a favorable financial environment for the integrated development of rural industries. Governments should expedite the construction of rural networks and financial infrastructure in underdeveloped areas, intensify the promotion and educational initiatives of digital inclusive financial services, and prioritize “urban-led rural development” and “urban-rural integration” strategies. This aims to optimize the allocation of agricultural resources, restructure rural industries, expand agricultural value chains, explore novel multi-functional agricultural formats, innovate agricultural technologies, and enhance agriculture’s adaptability to complex and ever-changing natural and social environments.

Finally, tailor-made rural industry integration and digital inclusive finance policies based on local development characteristics are needed. The empirical results indicate regional disparities and disparities in agricultural development within Zhejiang. In response to these findings, targeted support policies should be introduced. Specifically, these policies should focus on enhancing the digital inclusive finance infrastructure in Southwest Zhejiang, enriching the diversity of digital financial products, and promoting digital inclusive finance in this region. The first-tier agricultural development regions should share successful rural industry integration models and cases with third-tier regions. This transfer of knowledge and expertise could uplift the overall development and capabilities of the agricultural and forestry industry in third-tier regions.

## Supporting information

S1 Data (XLS)
